# How Many Parameters Does It Take to Describe Disease Tolerance?

**DOI:** 10.1371/journal.pbio.1002435

**Published:** 2016-04-18

**Authors:** Alexander Louie, Kyung Han Song, Alejandra Hotson, Ann Thomas Tate, David S. Schneider

**Affiliations:** 1 Department of Microbiology and Immunology, Stanford University, Stanford, California, United States of America; 2 Program in Immunology, Stanford University, Stanford, California, United States of America; 3 Department of Biology and Biochemistry, University of Houston, Houston, Texas, United States of America; Princeton University, UNITED STATES

## Abstract

The study of infectious disease has been aided by model organisms, which have helped to elucidate molecular mechanisms and contributed to the development of new treatments; however, the lack of a conceptual framework for unifying findings across models, combined with host variability, has impeded progress and translation. Here, we fill this gap with a simple graphical and mathematical framework to study disease tolerance, the dose response curve relating health to microbe load; this approach helped uncover parameters that were previously overlooked. Using a model experimental system in which we challenged *Drosophila melanogaster* with the pathogen *Listeria monocytogenes*, we tested this framework, finding that microbe growth, the immune response, and disease tolerance were all well represented by sigmoid models. As we altered the system by varying host or pathogen genetics, disease tolerance varied, as we would expect if it was indeed governed by parameters controlling the sensitivity of the system (the number of bacteria required to trigger a response) and maximal effect size according to a logistic equation. Though either the pathogen or host immune response or both together could theoretically be the proximal cause of pathology that killed the flies, we found that the pathogen, but not the immune response, drove damage in this model. With this new understanding of the circuitry controlling disease tolerance, we can now propose better ways of choosing, combining, and developing treatments.

## Abstract 2

Experiments using fruit flies infected with *Listeria monocytogenes* show that changes in the shape of a disease tolerance curve can reveal the source of pathology for an infectious system.

## Author Summary

It is an intuitive assumption that the severity of symptoms suffered during an infection must be linked to pathogen loads. However, the dose–response relationship explaining how health varies with respect to pathogen load is non-linear and can be described as a “disease tolerance curve;” this relationship can vary in response to the genetic properties of the host or pathogen as well as environmental conditions. We studied what changes in the shape of this curve can teach us about the underlying circuitry of the immune response. Using a model system in which we infected fruit flies with the bacterial pathogen *Listeria monocytogenes*, we observed an S-shaped disease tolerance curve. This type of curve can be described by three or four parameters in a standard manner, which allowed us to develop a simple mathematical model to explain how the curve is expected to change shape as the immune response changes. After observing the variation in curve shape due to host and pathogen genetic variation, we conclude that the damage caused by *Listeria* infection does not result from an over-exuberant immune response but rather is caused more directly by the pathogen.

## Introduction

The clinical goal of treating infectious diseases is to reduce the levels of sickness experienced by infected hosts. One approach to studying this problem is to quantitate illness by correlating the dose response of pathology to pathogen load; graphs like these are called “disease tolerance curves” [1–6]. We argue that the shape of the dose response curves and the underlying mathematical functions producing these curves can teach us how to alter the health-by-microbe relationship. For example, if disease tolerance curves are linear, we need only discover the molecular mechanisms that control the curves’ slopes. If these curves have a more complex shape—for example, a sigmoid shape—then we will need to measure more variables.

Since there are not universally agreed upon definitions for many of the words we use to describe the immune response and health, we start by defining the following terms: vigor, resistance, resilience, and tolerance. “Vigor” is the health of an uninfected individual, and “resistance” describes the host’s ability to limit microbe load [1–6]. We use “resilience” to describe the ability of infected hosts to return to their original healthy condition, much in the same way the word is used to describe the way a perturbed ecological system returns to its origin [7,8]. For example, a resilient host may suffer from an infection but would easily bounce back to health, whereas infection in a non-resilient host might lead to permanent disability or death. We apply the term resilience to an infected individual because we can follow the path that individual takes from health through sickness and back. “Disease tolerance” is the dose response curve summarizing the pathogen loads that are required to produce a range of host responses. Disease tolerance differs from resilience in that tolerance is an emergent property of populations, while resilience is a property of an individual; disease tolerance measures how resilience changes across a population as pathogen load is varied. Disease tolerance, by definition, cannot be measured in a single individual [9]. The model described in this paper shows how these distinctions between resilience of individuals and tolerance of populations disappear once we understand infection dynamics to the point that we can predict how a population would behave knowing the properties of an individual.

To study disease tolerance we used a simple infection system in which we injected a pathogen (*Listeria monocytogenes*) into a fruit fly (*Drosophila melanogaster*) [10–14]. These *L*. *monocytogenes*-infected flies suffer from a variety of ailments, including alterations in their circadian rhythm, cold-coma recovery, climbing ability, feeding behavior, metabolism, and death [12,15–17]. To determine the disease tolerance curve, we plot the survival rates of the infected flies against microbe loads measured 2 days post infection (DPI). We used this system because it allowed us to measure large numbers of samples and to take advantage of the genetic tools and the understanding of innate immunity available for this model organism.

Our immediate goal is to define the mechanisms controlling tolerance. We start with a simple mathematical model representing feedbacks between microbe growth, the immune response, damage, and health, modeling each using logistic equations. We then measured the ground-truth of these models using a *D*. *melanogaster/L*. *monocytogenes* infection that can lead to lethal outcomes. We experimentally measured microbe growth, the immune response, and disease tolerance and found that each of them was well described by sigmoid curves, suggesting that we need at least three variables to describe each. In the case of tolerance, this meant that we had to measure host vigor, the number of bacteria required to injure the host, and the maximum death rate achieved by the fly. In the case of antimicrobial peptide (AMP) transcript production, we measured basal levels, the number of bacteria required to induce transcripts to 50% of their maximum value, and the maximum transcript level. We examined how alteration of the values of the parameters in the model changed the output of the model to distinguish between changes that we might see if microbes or the immune response were the principal mediators of damage. Upon testing a variety of *D*. *melanogaster* natural variants and mutants along with some *L*. *monocytogenes* mutants, we concluded that, in this system, the bacteria and not the immune response is responsible for the damage caused by the infection. When we monitored how disease tolerance changed as we varied host or pathogen properties, we found the tolerance curve changed shape in the manner predicted by the model; for example, the number of bacteria required to damage health changed, while the vigor and maximum death rate remained constant. We demonstrate that this dual approach of measuring and modeling full-length disease tolerance curves can reveal previously unrecognized parameters controlling disease tolerance.

## Results

**Fig 1:**
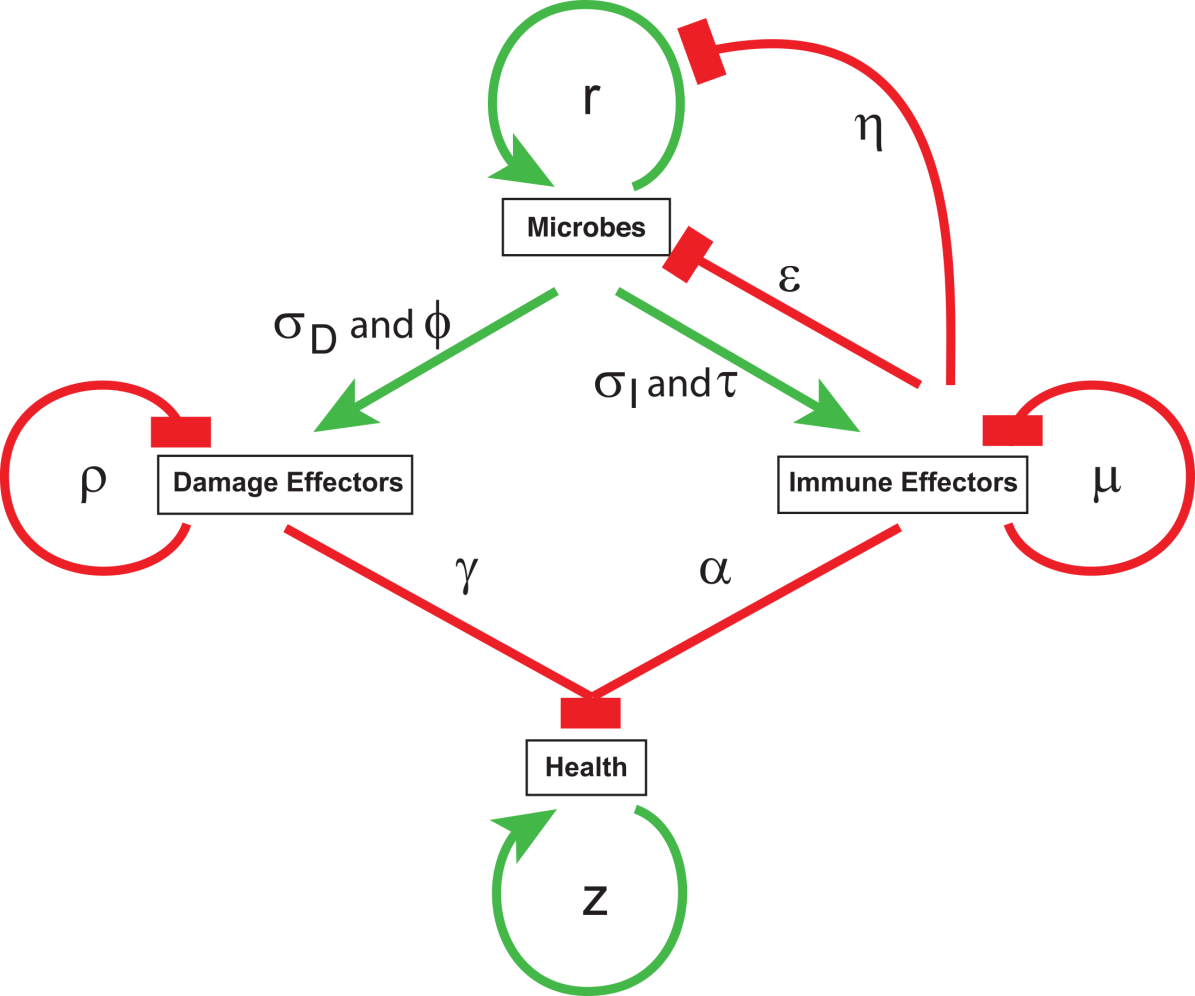
Visualizing infections in individuals, in pure lines and diverse populations. (A) A simple model in which microbes induce an immune response, which in turn limits microbe growth and kills the microbes. Both microbes and immune effectors can cause damage in this model.

We started by making a graphical model to explain four parameters: microbe load, the immune response, microbe-induced damage, and health (Fig 1). We modeled the immune response so that it could both limit microbe growth and kill microbes. We previously found that *Mycobacterium marinum* infected flies waste upon infection and predicted that the day they died they would have exhausted their glycogen and fat stores [18,19]. We also found that *L*. *monocytogenes* infected flies waste during infection [15]; to explain death, we used these empirical data to imagine that there was a store of “health” that could be depleted by the infection. In our model, both the immune response and the microbes can cause damage, which depletes health and increases the death rate. Immunopathology directly affects health, while microbes secrete damage effectors that impact health.

We built a simple mathematical model based on the graphical model largely using sigmoid functions to define each parameter (see Experimental Procedures) to help define our assumptions about the mechanisms governing disease tolerance. This was an obvious choice for microbe growth and the immune response, as these equations are well described in classical mathematical models of infections [20]. We used the same mathematical function to describe microbe-induced damage, since we anticipate that the shape of the curve is due to some enzymatic rate-limiting step that will likely follow sigmoid Michaelis-Menten kinetics [21,22].

To determine the ground-truth of this model, we measured the behavior of microbes, the immune response, and health in a model system in which we inject *L*. *monocytogenes* into the body cavity of *D*. *melanogaster*. We chose this model infection because it is simple to obtain the measurements we need to define resistance and disease tolerance and because we have a collection of host mutants that we know affect resistance and tolerance to *L*. *monocytogenes* [10–12]. We can readily measure microbe loads by homogenizing whole flies at any point in the infection to plate them to determine colony-forming units (CFU). We can determine the health of the infected flies by measuring the median time to death following infection.

**Fig 2:**
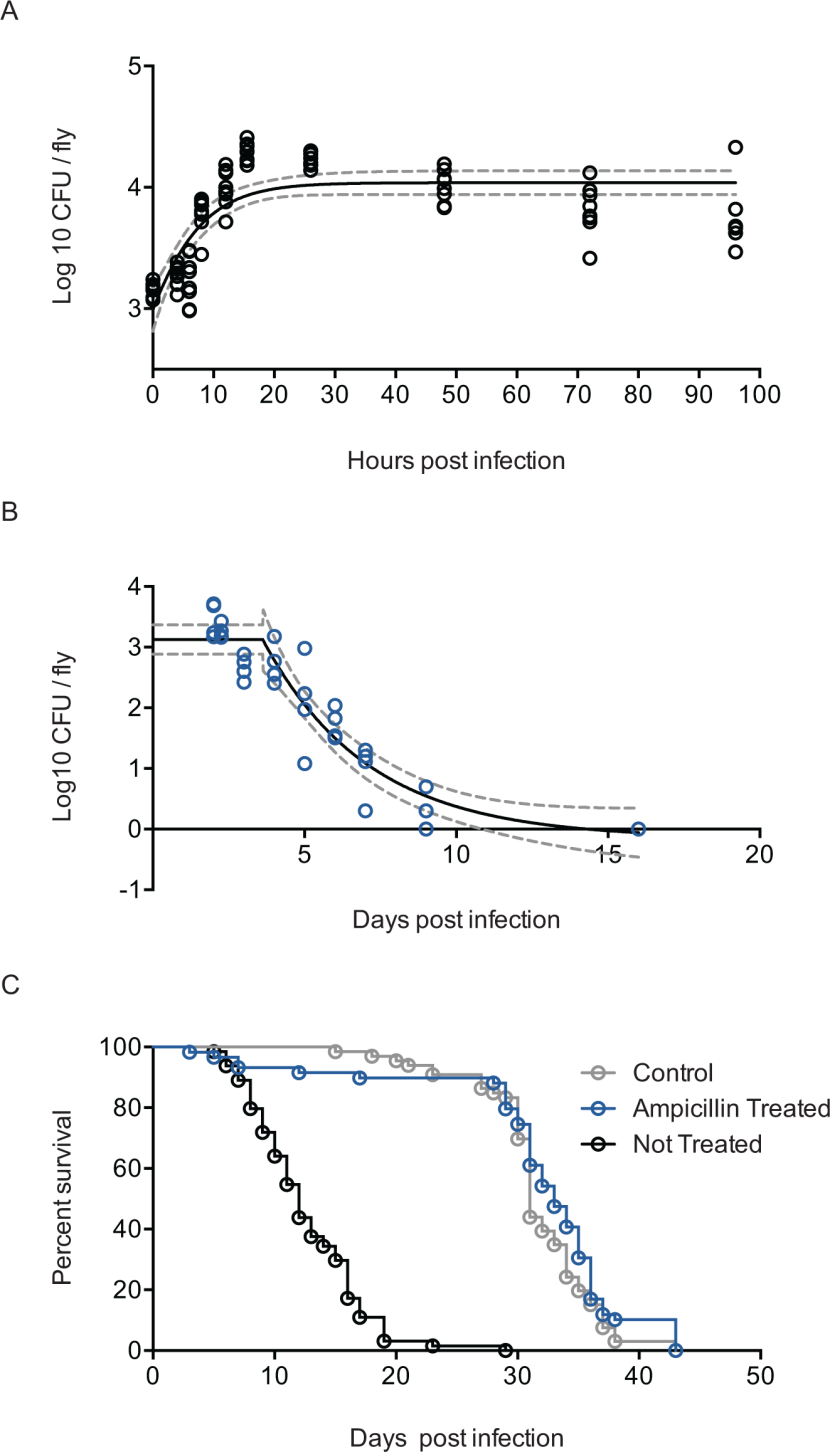
*Listeria monocytogenes* growth characteristics in the fly. (A) *L*. *monocytogenes* growth dynamics during infection. w^1118^ flies were injected with 1,000 CFUs. Individual flies were homogenized and plated, and bacterial colonies were counted at each time point. The data are fit with a logistic curve with a maximal growth ceiling of approximately 17,000 bacteria and a growth rate of 0.1659/h. The dotted lines indicate the 95% confidence interval. (B) *L*. *monocytogenes* dynamics during antibiotic treatment. Flies were injected with 100 CFUs and treated with ampicillin. The data are fit with a plateau followed by a one-phase decay with a half-life of 2.4 d. The dotted lines indicate the 95% confidence interval. (C) Survival curves for treated and untreated flies demonstrate that antibiotic treatment led to recovery of the flies, which lived as long as wounding control flies. Data are reported in S1 Data.

When *L*. *monocytogenes* is injected into flies that are incubated at 29°C, the resulting bacterial growth is best described as logistic (Fig 2A, logistic adjusted r^2^ = 0.594, adjusted linear and adjusted exponential r^2^ < 0.1) with a maximum growth rate of 0.1656 log10 (CFU)/h. *L*. *monocytogenes* loads reach a plateau at 24 h post infection.

We wanted to define the relationship between AMP levels to a given microbe load to understand how many parameters we need to use to measure the immune response. Because bacteria grow rapidly to reach their plateau, and wounding flies to introduce bacteria causes an immune response on its own, we reasoned it would be difficult to measure the immune responses at intermediate microbe loads by simply injecting flies with different amounts of microbes. Instead, we took flies that had high levels of bacteria and slowly cured them of the infection so that we could measure the relationship between AMP transcripts and microbe load essentially at steady state. The fly immune response cannot clear *L*. *monocytogenes* on its own; therefore, to clear the bacteria we transferred infected flies to fly media containing 1 mg/ml of ampicillin 2 DPI. We observed that *L*. *monocytogenes* clearance followed exponential decay dynamics (Fig 2B, adjusted r^2^ = 0.7140). *L*. *monocytogenes* infection in the fly decreases survival, but ampicillin-treated flies lived just long as uninfected flies (Fig 2C).

Having developed a method to slowly decrease microbe loads, we performed the following experiment: We injected one set of flies with phosphate buffered saline (PBS), fed them with ampicillin, and followed them regularly to serve as wounding, aging, and antibiotic treatment controls. We took a second set of flies and injected *L*. *monocytogenes*. This group was split in two; one portion was fed ampicillin to clear the microbes, and the other was not. We collected flies for plating to determine microbe loads, and for RNA preparation to run a microarray at regular intervals (S1 Fig).

**Fig 3:**
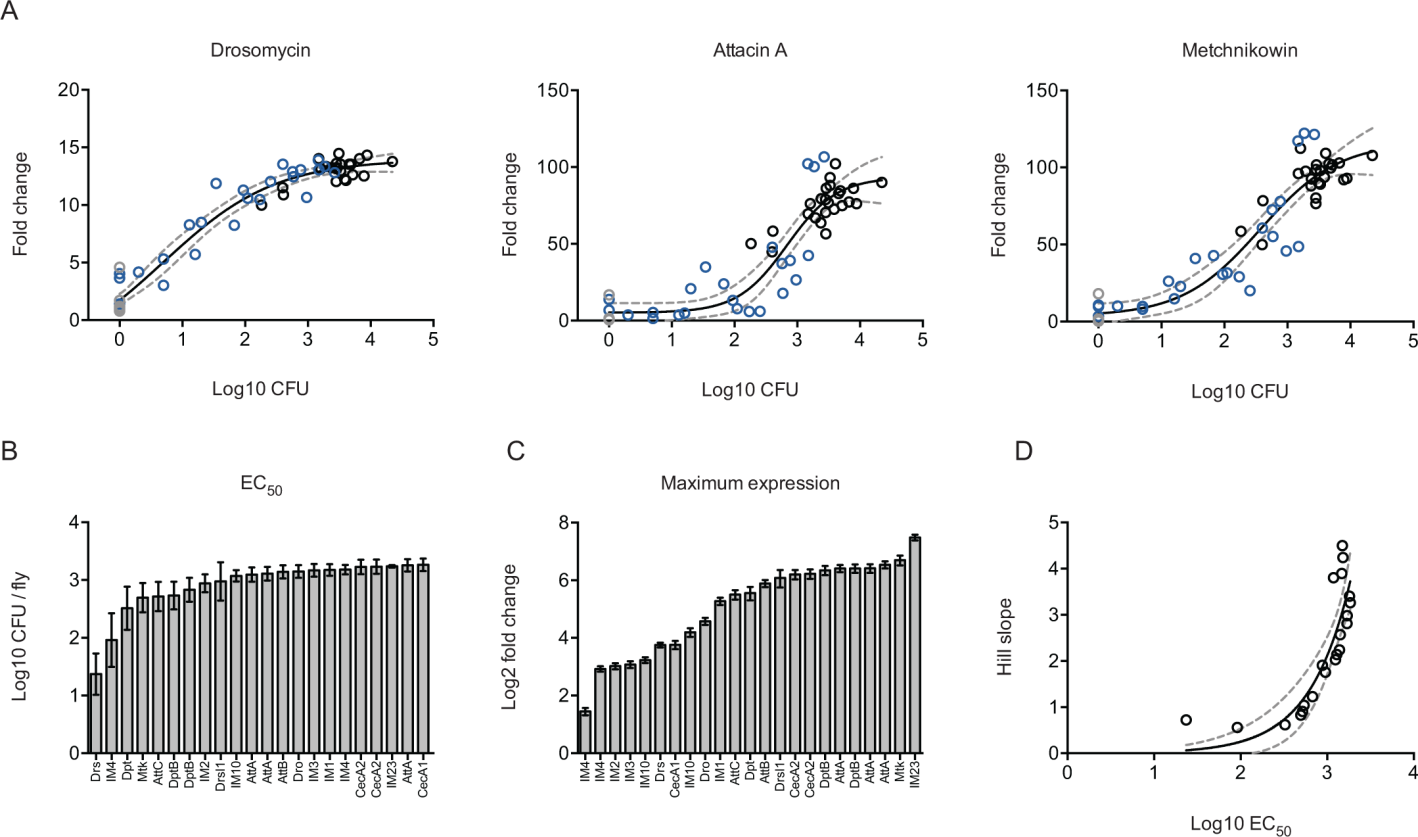
Antimicrobial peptide expression dynamics. (A) AMP dose response curve. Expression of AMPs was determined by microarray. Fold change is plotted relative to log microbe load (grey: uninfected control, black: infected, no treatment, red: infected, ampicillin treatment). The data are fit with a four-parameter sigmoid model. Adjusted r^2^: *Drosomycin* = 0.9521, *Attacin A* = 0.8509, *Cecropin A* = 0.8933. The dotted lines indicate the 95% confidence interval. (B) EC_50_ of antimicrobial peptides. Error bars mark the 95% confidence interval. (C) Maximum expression level of antimicrobial peptides. Error bars mark the 95% confidence interval. (D) Relationship between Hill slope and EC_50_. The data are fit with an exponential growth function with an adjusted r^2^ = 0.5126. Data are reported in S1 Data.

We analyzed the expression of the known and presumed AMPs with respect to microbe loads and found that AMPs are expressed according to a sigmoid relationship with respect to microbe load. We plotted the expression of AMPs to CFU levels in the fly and found the relationship between AMP transcription and microbe load is best fit by a four-parameter sigmoid (Fig 3A). Accordingly, each AMP transcript has a baseline (bottom plateau), maximal concentration (top plateau), effective bacterial concentration producing a half maximal effective concentration (EC_50_), and slope. We observe that the AMPs expressed during infection differ in their EC_50_ (Fig 3B) over a range of about 100-fold (20–2,000 CFUs). AMP expression does not continue to increase as microbe number increases past a certain point, and each AMP transcript reaches a characteristic plateau. Each AMP transcript can thus be characterized by its maximum expression in relation to its baseline, and this relationship varies over an 8-fold range between AMPs. We observed no significant relationship between EC_50_ and maximum expression, suggesting that EC_50_ and maximum expression can vary independently of each other. We found an exponential relationship between Hill slope and EC_50_ (Fig 3D), suggesting that AMPs requiring large numbers of bacteria for induction turn on in a switch-like manner, while those turned on at low microbe loads are turned on gradually. This gives us insight into the tuning of the fly’s immune response; overall, AMPs will be turned on gradually at microbe loads below 100 bacteria per fly but will rapidly increase as loads pass 1,000 bacteria per fly.

Though AMPs had a rather simple sigmoid relationship with microbe load, this was not the case for many other genes modulated during infection. Some of these other gene expression patterns showed hysteresis, by which we mean that gene expression depends upon past conditions and not only the immediate conditions. For example, gene expression for these hysteretic transcripts had one pattern as the fly moved from health to sickness and another as the fly returned from sickness to health. This hysteresis can be useful because it helps us define different states of the infection. To visualize the “disease space” traversed by infected flies and to identify the different states of the infection process, we used topological data analysis (TDA). It is common practice when clustering data to plot the data using some sort of tree structure, as is done with hierarchical clustering or spade analyses [23,24]. This makes good sense when dealing with data for a developing system in which the animal or cell enters a new state, but it is problematic when dealing with a system that returns to its original state; resilient systems should not be fit by a tree and are better described by loops. Topological data analysis is sensitive to the topology of the data and will not arbitrarily linearize a loop and then force it to fit a tree [25,26]. Instead, the analysis simply clusters related data points, represented as nodes on a network graph, and the shape of that graph reveals the connections between the time points (for recent examples see [27–29]). In the case of a resilient system, such as flies recovering from an infection, we find this graph forms a loop.

**Fig 4:**
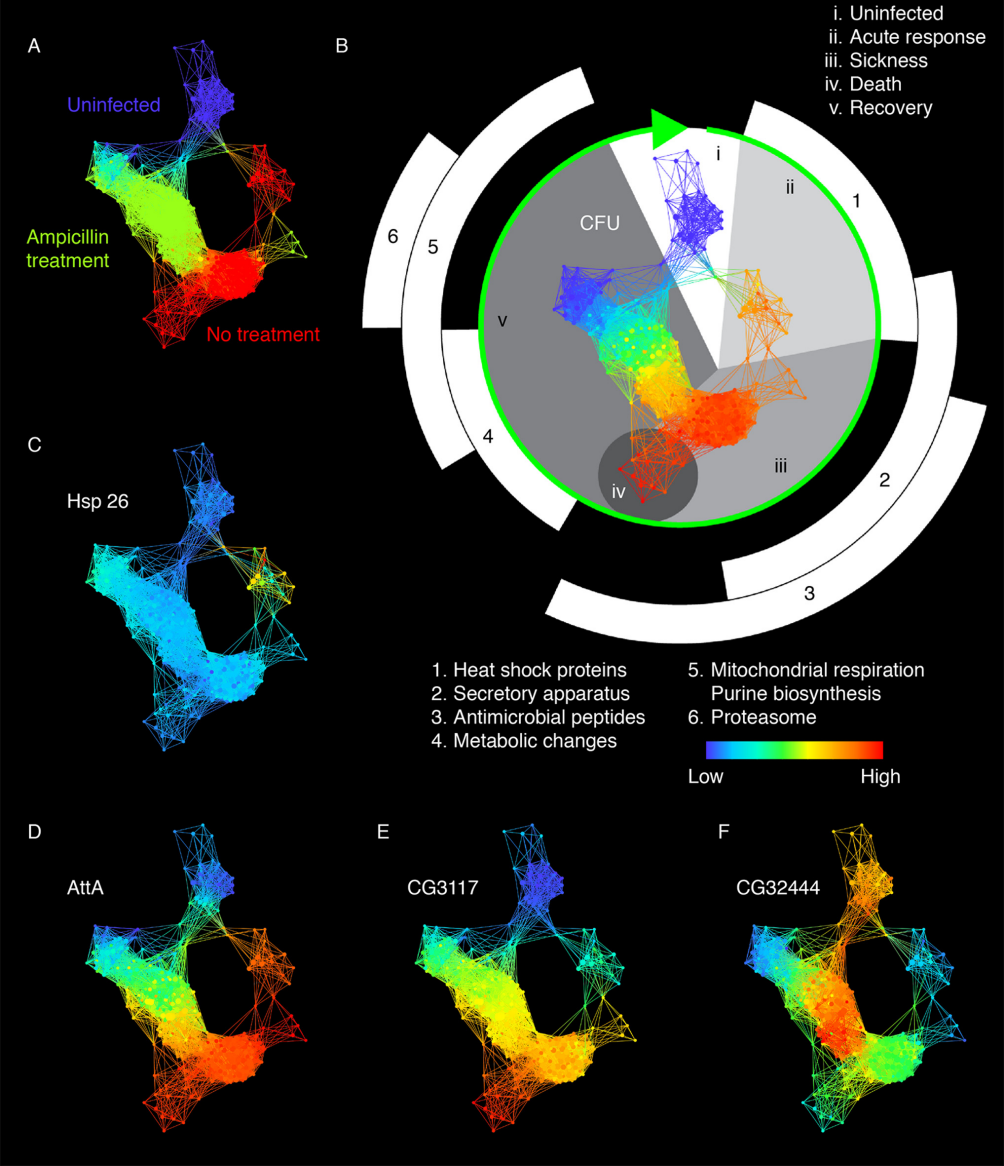
Disease space analysis of infected and recovering flies. (A) Infection map colored by treatment groups. The network was built using Ayasdi Core (data from S1 Table). Samples with similar expression patterns are binned together. Nodes are bins of individual samples. Bins containing the same sample are connected by edges. Cyan is the overlap of uninfected and no treatment groups, and orange is the overlap of ampicillin treatment and no treatment groups. (B) Infection map colored by CFU. The green arrow marks disease progression. Pathways of upregulated genes are numbered. Phases of infection are indicated by Roman numerals. (C) Infection map colored by *heat shock protein 26*. (D) Infection map colored by *Attacin A*. (E) Infection map colored by *CG3117* (peptidase and death gene). (F) Infection map colored by *CG32444* (aldose-1 epimerase and recovery gene).

Fig 4 shows the TDA graph we built to describe our data, and 4A describes the three basic treatment groups in the dataset (uninfected, infected, and antibiotic-treated). In Fig 4B, the phase curve is colored by CFU (blue indicates low and red indicate high microbe loads). The green arrow indicates progression from health to infection and back, moving clockwise around the figure. Uninfected flies are at the top of the figure, and the figure progresses through acutely wounded flies, sick flies, and on to recovering flies, which link back to the original uninfected flies. Infection was initiated by the injection of 100 CFU, and *L*. *monocytogenes* levels increase following this. The path followed by moribund flies deviates from recovering flies to form a spur on the loop (marked as section iv). Flies that received ampicillin decrease in CFUs, and flies that have low CFUs loop back to overlap with uninfected controls.

To determine which genes show altered expression in different parts of the disease map, we performed fuzzy c-means clustering on this dataset to identify genes with similar expression patterns (S2 Fig, S2 Table). We then performed gene ontology analysis on each cluster to identify characteristic physiological changes within the cluster (S3 Table) and then asked about the expression patterns of these genes on the TDA graph. Acute stress response genes, such as heat shock protein 26, were activated immediately after injection and then quickly returned to baseline levels (Fig 4B and 4C). AMP expression follows CFU (Fig 4B and 4D). We identified groups of genes that are highly expressed by flies in the spur approaching death (Fig 4B and 4E) and recovering from infection (Fig 4B and 4F). The transcripts for these “morbidity genes” (clusters 1, 2, 11, 12, 22, 35, 43) continue to increase even when microbe levels plateau. Gene ontology analysis of fuzzy c-means clusters containing these morbidity genes did not yield significantly enriched terms. Among the top 50 differentially expressed morbidity genes, *Bteb2* and *run* are predicted to encode proteins with DNA binding activity. *CG3117* has predicted protease activity, and *diedel* is a putative negative regulator of JAK/STAT signaling. By contrast, the recovery genes (clusters 18, 20, 23, 25, 29, 30) are enriched for sugar and fatty acid metabolism genes that are turned up above baseline upon recovery.

### The Shape of a Disease Tolerance Curve

**Fig 5:**
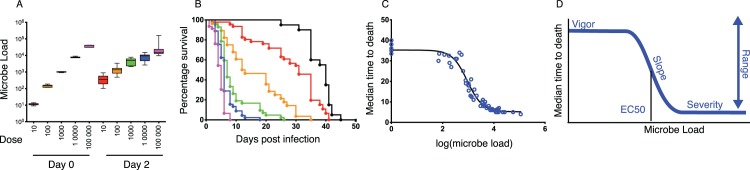
Disease-tolerance curves are sigmoid. (A) Dose-dependent growth of *L*. *monocytogenes* during infection. Wild-type flies injected with 10–100,000 *L*. *monocytogenes* were homogenized and then plated 2 d post-infection to determine microbe loads. (B) Dose-dependent survival of *L*. *monocytogenes*-infected flies. Kaplan-Meier curves were plotted for flies injected with 10–100,000 *L*. *monocytogenes*. (C) Disease-tolerance curve. Pairs of microbe load and survival data for 63 microbe load/MTD pairs were plotted. This curve was fit with a four-parameter sigmoid model (r^2^ > 0.96). (D) A cartoon showing the parameters used to describe a sigmoid disease-tolerance curve including vigor, slope, EC_50_ (sensitivity), and maximal severity, as well as the measurement of phenotypic range. Data are reported in S2 Data.

To measure a disease tolerance curve, we recorded the response of the host to a broad range of initial pathogen doses. We did this by injecting *L*. *monocytogenes* into flies over a range of ten to 100,000 bacteria and allowing the infected flies to die. We injected *L*. *monocytogenes* into the hemocoel of flies and monitored bacterial numbers 2 DPI to measure the ability of the fly to resist microbe growth when challenged with a range of infection intensities (Fig 5). We determined the median time to death (MTD) for each inoculum and used this time as a measurement of health (Fig 5B). Plotting microbe load versus MTD produced a curve that was readily fit by a four-parameter logistic sigmoid model (r^2^ > 0.96) (Fig 5C).

Occasionally, tolerance curves are fit with mathematical functions, but the reason these functions are chosen is that they fit the data and not that they are dissected for further biological insights [5,30–32]. More typically, tolerance is visualized as a linear system, which requires just two parameters, vigor and slope [5,11]. In contrast, our sigmoid model provides four parameters (Fig 5D). In addition to vigor and slope used to describe a line, the sigmoid model adds the parameters of EC_50_ and maximum effect. The EC_50_ of the system is the number of microbes present at day 2 that cause a 50% change in MTD. Maximum effect is defined by the sigmoid’s asymptotic tail at high microbe loads. This defines a maximum death rate, suggesting that there is a previously unrecognized rate-limiting step for death.

**Fig 6:**
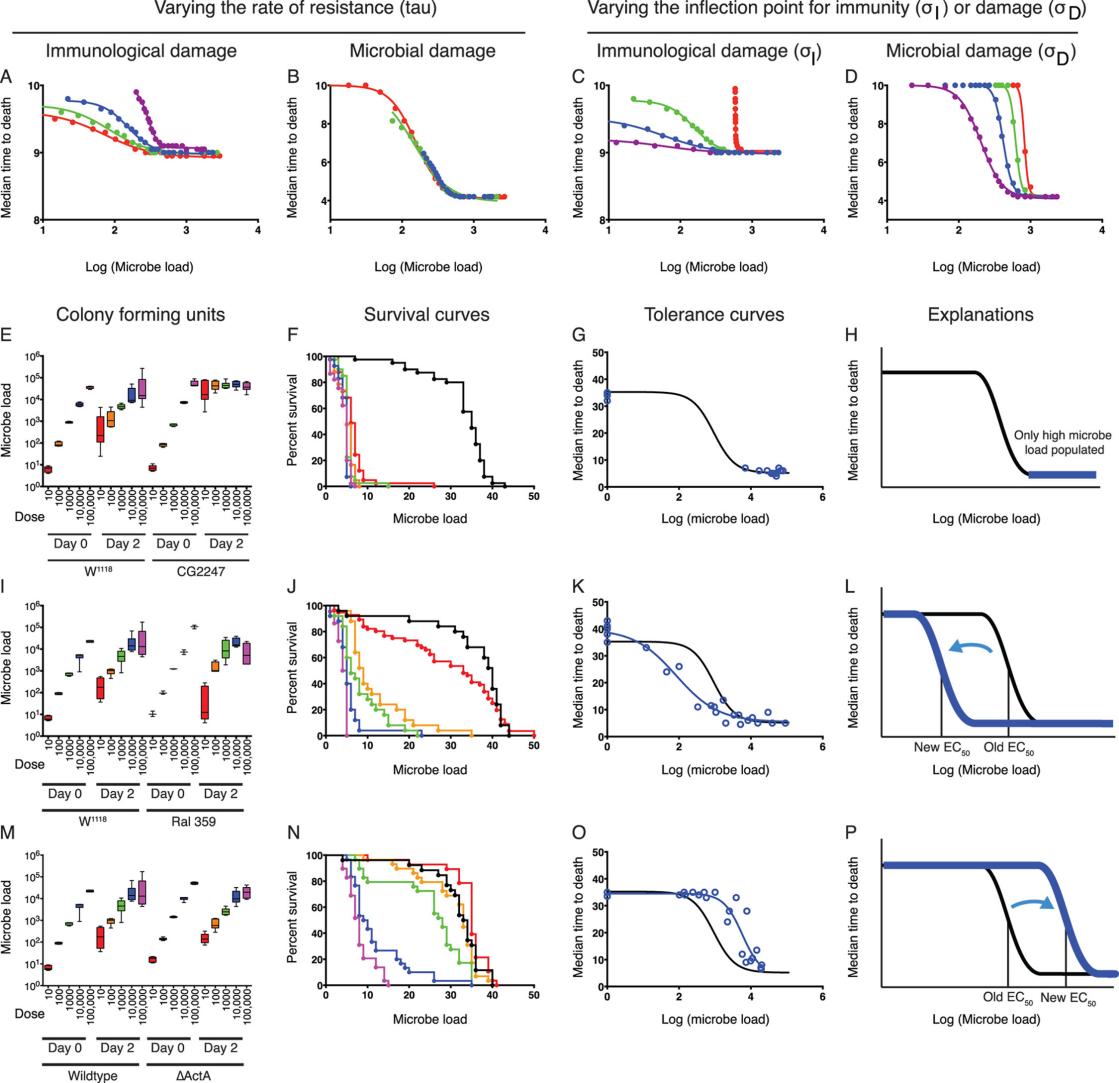
Predicted and observed variation of tolerance curves. (A–D) Predicted changes in infection tolerance curves as the rate of immune effector production (τ) and the inflection points for microbe-induced immunity or damage are altered in the immunological damage model (α only) and the bacterial damage model (ϕ only). Each line is the sigmoid fit of values computed by the model. The colors represent the value of the altered parameter, moving from violet to blue to green to red as the value increases. (E–H) Tolerance curve of a resistance-deficient fly strain (*CG2247*) infected with wild-type *L*. *monocytogenes*. (I–L) Tolerance curve of a natural variant *D*. *melanogaster* strain infected with wild-type *L*. *monocytogenes*. (M–P) Tolerance curve of *L*. *monocytogenes ΔactA* mutants injected into w^1118^ control flies, (M–P). Microbe loads are recorded in (E), (I), and (M). Survival curves are recorded in (F), (J), and (N). Tolerance curves for the condition being tested are reported in (G), (K), and (O), with the corresponding data points in blue. The tolerance curve for w^1118^ is shown in black without data points. Panels (H), (L), and (P) show illustrations of the changes in parameters seen in the tolerance curves. Data are reported in S2 Data.

In the model shown in Fig 1A, depletion of health could be induced either by bacterial damage effectors or indirectly by self-harm caused by the resistance response. We modeled the two possibilities by observing how the shape of the curve changed as we altered the model such that either the resistance response or bacteria were the sole cause of damage. We concentrated on changes in the rate that the immune response was turned on (τ), and the inflection points for the relationships between microbe density and the rates of immune induction and microbe-induced damage induction (σ_I_ and σ_M_) (Fig 6A–6D, S4 Table). In the case in which resistance mechanisms drove damage (Fig 6A and 6C) and bacterial damage was set to zero, changes in τ or the inflection point for microbe-induced immunity caused shifts in both the EC_50_ and microbe loads. The effect is more extreme when the inoculum is far below the microbial carrying capacity, as this gives the immune response an opportunity to control microbe loads. This results in a reciprocal mechanistic link between resistance and tolerance in which one is always high when the other is low. In the model in which bacteria drive damage, a loss of resistance results in high microbe loads and health pegged at maximum severity (Fig 6B). Shifts in the EC_50_ of the bacterial damage-driven system, in which resistance-induced damage is set to zero, are caused by changes in the inflection point for bacterial-induced damage, as might be expected for differentially virulent strains of microbes or hosts that are better at neutralizing bacterial toxins (Fig 6D). In this second case, there is no mechanistic reciprocal link between resistance and tolerance, and the two vary independently of each other.

### Testing the Mathematical Model for Disease Tolerance

To test whether the shape of the disease tolerance curve in this host–pathogen pairing was driven by immunological damage or microbial damage, and to determine how the parameters of a sigmoid disease-tolerance curve varied, we measured the tolerance curves for a collection of *Drosophila* mutants that we previously showed differ in their resistance and tolerance defenses to *L*. *monocytogenes* [10–12]. We also tested natural variant flies from the *Drosophila* Genetics Reference Panel that were preselected because they showed extreme changes in their response to *L*. *monocytogenes* infections [33,34]. In addition, we tested *L*. *monocytogenes* mutants that altered pathogenesis in the fly [11]. These data supported the model in which bacteria caused damage in the system and did not support the immunological damage model.

A mutation in the *D*. *melanogaster* gene *CG2247* was found previously to reduce the fly’s ability to both survive an infection and control *L*. *monocytogenes* growth [11]; here, we found that at 2 DPI, even *CG2247* mutant flies injected with just ten bacteria had the same high microbe loads as flies injected with 100,000 bacteria, supporting the idea that these mutants alter resistance. This is visible in the tolerance curves, in which all of the points from infected mutant flies clustered on the bottom asymptote of the parental tolerance curve (Fig 6E–6H, S5 Table). An additional example of a fly strain with poor resistance is shown in the supplementary data (S3 Fig and S5 Table). Since flies suffering a loss of resistance showed no change in the maximum death rate of the infection, these results support the model in which bacteria are responsible for damage.

RNAseq analysis of *CG2247* mutant flies suggests a molecular mechanism for the observed phenotype. We observed that in wild-type flies, the transcripts encoding the enzymes required to generate a reactive oxygen response against *L*. *monocytogenes* dropped over the course of the infection but recovered upon antibiotic treatment (S6 Fig) [11,35]. In contrast, these transcripts dropped to 10-fold lower levels in *CG2247* mutants and did not recover to their original levels. We observed a reduction in the characteristic dark spots resulting from the action of this immune response (S6 Fig). We conclude that mutations in this gene disrupt the melanization immune response.

The disease-tolerance curve for the natural variant *D*. *melanogaster* strain, RAL 359, showed a reduction in the EC_50_ of the system without a change in resistance [33]. RAL 359 was as capable as our lab control strain w^1118^ in controlling *L*. *monocytogenes* growth; however, low doses of the microbe had a larger effect on health in this strain than in the control. This shifted the EC_50_ from 922 to 83 colony-forming units per fly (Fig 6I–6L and S5 Table). The vigor and maximum death rate of these two strains was similar. This shift in EC_50_ was a common phenotype, as shown in S4 and S5 Figs.

Changing the virulence of the *L*. *monocytogenes* also altered the EC_50_ of the system. Mutant *L*. *monocytogenes* lacking the virulence factors actin assembly-inducing protein (ActA) or listeriolysin O (LLO) had previously been shown to kill flies more slowly than wild-type *L*. *monocytogenes* (Fig 5C versus Fig 6O) [11]. The tolerance curves for flies infected with an *ΔactA* mutant showed a shift in the EC_50_ from 922 to 5,753 (Fig 6M–6P and S5 Table). We observed a ceiling for the number of *L*. *monocytogenes* that can be maintained in a wild-type infected fly (Figs 2A and 5A) and hypothesize that this is defined by the number of phagocytes in the fly that provide a niche for *L*. *monocytogenes* growth [13]. The *ΔactA* mutant bacteria approached this limit before they induced maximal pathology and, thus, we did not observe a low asymptotic tail. *Δhly* mutants produced a similar result to *ΔactA* mutants, only more extreme (S4 Fig).

## Discussion

As a field, we can generate complex network diagrams showing how the parts of the immune system communicate with each other and identify key signaling pathways and nodes [36,37]. Though this lets us predict that the system will fail when we remove a necessary component of the network, evolution and medicine work by subtler means, for example, by changing rate constants in addition to deleting entire pathways. To predict how evolution or medicine will change an immune response, we need to identify mathematical functions that accurately describe the behavior of that process so that we can understand all of the parameters that control the behavior.

*L*. *monocytogenes* growth in the fly follows logistic kinetics; the variation of these curves observed in natural fly variants suggests that microbes are controlled by separable parameters defining the growth rate and carrying capacity of the host [34]. It seems reasonable that this is not a property limited to *L*. *monocytogenes* growing in the fly and that it will be true for many pathogens, especially considering that this is a near textbook description of microbial growth [20,38,39]. Both of these properties should be under natural selection, but studies of infections in *D*. *melanogaster* typically do not measure microbe growth at all and, when they do, they measure only one time point, conflating growth rates with carrying capacities. The fly is studied because it is easily manipulated and has a relatively simple immune response, but despite decades of work, the field has been underestimating the number of dimensions in which natural variation can change the immune response. Thinking about this generally, it raises the question of whether there might be separate host mechanisms that control microbe growth rates versus ceilings and, thus, diseases might result from a defect in either of these mechanisms. To study these different disease mechanisms, we must ensure that our experiments let us observe the phenomenon.

The AMP response in the fly is under the control of two signaling pathways, Toll and imd [36,40]. The classical descriptions of these pathways argue that the pathways show specificity for different elicitors; a problem with the experiments supporting this model is that the experiments did not correlate response to elicitor levels, and most experiments tested just one arbitrarily determined time point [41,42]. We find that AMP induction is described well by a four-parameter sigmoid function. This means that there are parameters describing the basal level, EC_50_, maximum expression level, and slope, which may all vary independently of each other; that we don’t see a correlation between maximal gene induction and EC_50_ supports this hypothesis. If the field’s experiments measure gene expression at just one time point post infection and do not correlate this to microbe loads, we cannot learn which of the three induction parameters are changing. Thus, though it is clear that the Toll and imd signaling pathways, as well as sex, age, the environment, and evolution, can change the immune response, we don’t have a good definition of specificity in this system. Now that we understand that there are new variables to explain, we need to explain this from an evolutionary perspective; for example, what selective pressures cause changes in the EC_50_ of gene expression of an AMP rather than the maximal gene expression or slope? Will a host benefit from a quick induction of antimicrobials at low microbe loads, or should it invest in an enormous response as microbe loads climb higher?

We found that health was correlated with microbe load according to a four-parameter sigmoid function. As described above for microbe growth and immune induction, past experiments looking at health outputs in model infections are problematic because they don’t measure the full shape of the response curve and don’t report where an experiment is performed on the curve. Perhaps it should have been obvious that microbes would have a growth ceiling and AMPs would have a maximum expression level; classic infectious disease modeling texts start with chapters describing these functions [20,43]. However, one thing these books don’t do is link pathogen growth and the immune response to health. When we examine this relationship, we find that we can make simple models that predict the presence of more parameters than we’ve been studying. For example, the implication of a sigmoid response for health suggests that there is a rate-limiting step for death and that death has a maximum rate. This means that it should be possible not only to change the EC_50_ of the system to reduce the rate at which damage occurs, but we should also be able to increase the health reservoir of a host so that it has high stamina and will be able to suffer damage for longer periods of time.

The underlying problem with the field’s experimental characterization of immune responses is that we focus on when responses are induced in terms of time and not why they are induced in terms of the quantity of inducer. The result is that we systematically overlook and conflate control mechanisms. By correlating effects to effectors, we can better understand microbial pathogenesis.

### Disease Spaces

The disease space analysis of recovering and dying flies reveals that far more is going on in these sick animals than AMP gene expression, and it lets us organize these events with a simple map. A commonly used time point for gene expression studies in *Drosophila* is 6 h post infection [44,45]; this is a particularly problematic time point because it combines transient expression of heat shock genes with expression of antimicrobials. Flies fated to die have a progressing gene signature in which a set of transcripts is increased without a corresponding increase in microbe load. We anticipate that one will need to measure the course of pathogenic infections to identify these genes, rather than follow microbes that simply elicit an immune response but do not kill wild-type flies (for example *Escherichia coli* or *Micrococcus luteus* [40,46]. Just as each pathogen causes a different disease in humans, we expect that there will be a range of pathologies caused by insect pathogens and do not assume that these *L*. *monocytogenes*-induced genes are the only morbidity genes in the fly. The nature of this particular death response is unclear, as the identity of the genes does not provide obvious clues. In contrast, recovering flies have a unique gene expression signature that suggests function; for example, enzymes in biosynthesis and energy metabolism are repressed during infection but pop up hysteretically as microbes are removed, suggesting the induction of a recovery response that rebuilds damaged tissues.

### Why Does Shape Matter?

The shapes of tolerance curves are important because they will define which medications can be used to improve the health of an infected patient depending upon their position on the sigmoid curve. Here is a concrete example: If we think about the dose response curve to sepsis in humans as a linear response, then any drug that limits damage will help every patient. We come to a different conclusion if we consider a sigmoid relationship. Humans suffer from sepsis when relatively small numbers of microbes enter the blood, and very sick patients can have much higher levels of microbes. This suggests the sickest patients will be found on the asymptote of a sigmoid curve, where they will be suffering maximum pathological effects. Drugs that change the basal level of a response, EC_50_, or slope will not help these patients; they will only respond to drugs that manipulate maximal response levels. Thus, we need to understand the shape of these tolerance curves and where the patients lie on the curves to select appropriate treatments.

The discussion of tolerance in patients raises a tricky point. We’ve shown it is possible to measure a disease tolerance curve in a model system; this demonstrated the nonlinear nature of tolerance and suggested the existence of new dimensions we should follow when measuring disease outcomes. The problem is that this approach requires us to perform many more experiments than we do currently when analyzing phenotypes, and this approach is likely impossible to apply to most human infections for the following two reasons: First, disease tolerance is plotted using summary characteristics of an infection. For example, one might measure the maximum parasite load and minimum health for an infection [5]. This is accessible experimentally, but it is unethical to gather these data from a patient, as you need to treat the patient when they enter the clinic. In one rare case of HIV, infected patients’ tolerance curves have been recorded because these chronically infected patients are followed for years, but that approach isn’t going to be useful for acute infections [47]. Second, disease tolerance is a measurement of the behavior of a population and not an individual. The information we gather about an individual allows us to place a datum on a health by microbe graph, but we don’t know the shape of the curve that should be fit through that individual [9]; thus, we can’t easily determine which parameters need fixing in the sick patient. What we learn from model systems is that there are underlying rules that can be used to explain disease outcomes, and that these rules are nonlinear. By studying these rules in models, we can identify the molecular mechanisms corresponding to the mathematical parameters and then apply this knowledge to human biology by analogy. If we want to measure changes directly in human immune infections, we need to rely on descriptions of disease space, which are described in the accompanying paper [48].

## Experimental Procedures

### Fly Stocks and Husbandry

Flies were maintained on standard dextrose fly media (129.4 g dextrose, 7.4 g agar, 61.2 g corn meal, 32.4 g yeast, and 2.7 g tegosept per liter) at 25°C with 65% humidity and 12 h light/dark cycles. Shortly after eclosion, adult flies were collected into bottles containing dextrose fly media. At least 24 h post eclosion, adult flies were anesthetized with carbon dioxide, and males were sorted into groups of 20 and placed into vials containing standard dextrose fly media. Experiments were performed on flies 5–7 d post eclosion unless otherwise indicated. *CG2247* piggybac allele (BL18050), *Pcmt* piggybac allele (BL18398), *kenny* piggyback allele (BL11044), *CG7408* piggyback allele (BL19305), piggybac allele parental strain w^1118^ (BL6326), RAL 359 (BL28179), RAL 787 (BL28231), RAL 375 (BL25188), RAL 309 (BL28166), RAL 73 (BL28131), RAL 380 (BL25190), and RAL 821 (BL28243) strains were obtained from the Bloomington stock center.

### Injection

Bacteria were injected into flies essentially as described previously [10,12,14,49]. Flies were anaesthetized with carbon dioxide. A drawn glass needle carrying *L*. *monocytogenes* was used to pierce the cuticle on the ventrolateral side of the abdomen. A picospritzer III was used to inject 50 nl of liquid into the fly. Bacteria were delivered at different concentrations to produce injections of approximately 10, 100, 1,000, 10,000 or 100,000 CFU. Infectious doses were determined for each experiment by plating a subset of flies at time zero. Approximately 200–400 flies were used for each dose in the experiment to measure survival and to count colonies.

### Bacterial Strains and Culturing Conditions

All *L*. *monocytogenes* stocks: Wild type/mutant parental strain (10403S), *Δhly* (DP-L2161) [50], and *ΔactA* (DP-L3078) [51] were stored at -80°C in brain and heart infusion (BHI) broth containing 25% glycerol. To prepare *L*. *monocytogenes* for injection, bacteria were streaked onto Luria Bertani (LB) agar plates containing 100 ug/mL streptomycin and incubated at 37°C overnight. Single colonies of *L*. *monocytogenes* from the LB agar plate were used to inoculate 4 mL of brain and heart infusion (BHI) broth and incubated overnight at 37°C without shaking. Bacteria were removed from the incubator at log growth phase. Prior to injection, *L*. *monocytogenes* cultures were diluted to the desired optical density (OD) 600 in phosphate buffered saline (PBS) and stored on ice.

### Bacterial Plating

Single flies were homogenized in PBS using a motorized plastic pestle in 1.5 ml tubes. The supernatants were plated using an Autoplate spiral plater and counted using a Qcount automated counter. At least six samples were counted to determine the median number of bacteria for each inoculum. Bacteria were plated onto LB medium and incubated overnight at 37°C before counting.

### Survival Curve Analysis

200–400 flies were injected and checked daily to measure mortality for each inoculum. Flies were housed in vials containing 20–25 flies each.

### Repetitions

Each set of conditions was repeated at least three times; for example, an experiment for a mutant fly line would be set up independently on three different days to gather microbe load and survival data. Pairs of plating and survival data from these multiple experiments were all plotted on the same tolerance curve.

### RNA Isolation for Microarray

Flies were injected with 50 nl of Listeria (OD_600_ = 0.001, or approximately 100 CFUs) or left manipulated. Following injection, flies were placed in vials containing dextrose fly media and incubated at 29°C. Samples were separated into three groups: Moribund, recovering, and uninfected control. Moribund flies were infected, and samples were collected on the following DPI: 0.25, 1, 2, 2.25, 3, 4, 5, and 6. Recovering flies were infected and flipped onto dextrose fly media containing 1 mg/ml ampicillin 2 DPI. Recovering samples were collected on the following DPI: 2.25, 3, 4, 5, 6, 7, 9, and 16. Uninfected control flies were not infected but were flipped onto dextrose fly media containing 1 mg/ml ampicillin 2 dpi. Uninfected control samples were collected on the following dpi: 2.25, 3, and 9. At each of the indicated time points, groups of 20 flies were homogenized in TRIzol, and RNA was isolated using a standard TRIzol preparation. Additional flies were used to determine CFUs and monitor survival. Biological triplicates were obtained from three independent experiments. Quality of RNA was determined using a BioAnalyzer 2100. Samples were labeled using the Quick Amp Labeling Kit, One-Color (Agilent), and hybridized to 4x44K (V2) Drosophila Gene Expression Microarray (Agilent) using the Agilent Gene Expression Hybridization kit following the manufacturer’s protocol. Microarrays were scanned using an Agilent Technologies Scanner, and processed signal intensities were determined using Agilent’s Feature Extraction software. RNA quality assessment, labeling, hybridization, and microarray feature extraction were performed at the Stanford Functional Genomics Facility.

### Microarray Analysis

The microarray data were analyzed using Genespring v12.1 (Agilent). Microarrays were normalized to the 75th percentile. The median expression level on 0 dpi was set as the baseline. Prior to statistical analysis, low-quality spots were removed based on flag calls. To determine differential gene expression, one-way ANOVA was performed comparing all samples to 0 dpi. *P*-values were corrected by the Benjamini-Hochberg method. Genes with *p*-values <0.05 at any time point with a fold-change greater than 2 were categorized as differentially expressed. Differentially expressed genes were then clustered using Mfuzz [52].

### Topological Data Analysis

Only genes that were differentially expressed in moribund and recovering but not in uninfected control were used in the topological data analysis using the Ayasdi 3.0 software platform (ayasdi.com, Ayasdi Inc., Menlo Park, California). Nodes in the network represent clusters of samples of infected flies, and edges connect nodes that contain samples in common. Nodes are colored by the average value of their samples for the variables listed in the figure legends. TDA was used to map the way hosts loop through the disease space in an unsupervised fashion. Two types of parameters are needed to generate a topological analysis. First is a measurement/notion of similarity, called a metric, which measures the distance between two points in some space (usually between rows in the data). Second are lenses, which are real valued functions on the data points. Lenses are used to create overlapping bins in the dataset. Overlapping families of intervals are used to create overlapping bins in the data. Metrics are used with lenses to construct the Ayasdi 3.0 output. Multiple lenses can be used in each analysis. There are two parameters used in defining the bins. One is resolution, which determines the number of bins; higher resolution means more bins. The second is gain, which determines the degree of overlap of the intervals. Once the bins are constructed, we perform a clustering step on each bin, using single linkage clustering with a fixed heuristic for the choice of the scale parameter. This heuristic is described in [26]. This gives a family of clusters within the data, which may overlap. We built a network with one node for each such cluster and where we connect two nodes, if the corresponding clusters contain a data point in common. We used two types of lenses. The first type was lenses based on dimension reduction algorithms such as multidimensional scaling and nearest neighbor analyses; these helped analyze the data in an unsupervised manner. The second type was lenses based on the data alone, for example, the levels of antimicrobial gene expression or recovery gene expression. The gene expression markers helped us dissect the graphs using knowledge about the biology of the system. To build our map, we analyzed our dataset with samples as rows and genes as columns. We used the Variance Normalized Euclidean metric. We used the PCA coord1 and PCA coord2 lens as well as two data lenses: *CG32373* and *Fuca* (Resolution = 19, Gain = 6, and equalized was used for all lenses).

### Model Rationale and Methods

To evaluate the impact of within-host infection dynamics on host health curves, we created a compartmental model consisting of four ordinary differential equations for microbes (M), immune effectors (I), microbial damage effectors (D), and health (H).

$$\frac{dM}{dt}=(r-\eta I)M(k_{M}-M)-\varepsilon MI$$

$$\frac{dI}{dt}=\tau I(k_{I}-I)\left(\frac{k_{M}}{1+e^{-0.01*(M-\sigma_{I})}}\right)-\mu I$$

$$\frac{dD}{dt}=\varphi \left(1-\frac{D}{k_{D}}\right)\left(\frac{k_{D}}{1+e^{-0.01*(M-\sigma_{D})}}\right)-\rho D$$

$$\frac{dH}{dt}=zH(K_{H}-H)-(\alpha I+\gamma D)$$

Microbes grow in a sigmoid fashion at rate *r* as they deplete the host substrate, and immune effectors can either kill microbes outright at rate *ε* per effector or inhibit microbial growth at rate *η*. In these simulations, growth rate limitation was used as the main immune response. Two mechanisms contribute to immune effector production. Reflecting the sigmoid relationship between microbe density and AMP levels, immune effectors can increase in proportion to microbe density up to a maximal rate of τ*k_*M*_ until effector levels saturate immune pathway machinery (k_I_). The parameter σ_I_ reflects the microbe concentration at the inflection point for immune induction. Immune effectors decay at rate μ. Microbes secrete virulence factors and toxins at a maximal rate φ*k_D_, modulated by a sigmoidal relationship between damage factor production and microbe density to reflect a process like quorum sensing controls on virulence factor production. As with immunity, there is a carrying capacity imposed on damage effectors. This could reflect either negative feedbacks on further effector production at high effector densities or a bottleneck limiting flux through the system. The parameter σ_D_ reflects the microbe density that produces a half-maximal induction rate. Damage effectors degrade at rate ρ. Immunopathology and damage effectors deplete health (H) at rates γ and α, respectively, while hosts can recover health according to a sigmoidal relationship with current health (reflecting the difficulty of achieving recovery at low health due to organ failure and other catastrophe) at rate z. “Death” is called when health dips below 10% of maximum (k_H_). All simulations were conducted within a parameter space outlined in S4 Table and run in Matlab (v. 7.11.0) using the ode45 solver.

### Curve Fitting

The sigmoidal curves were fit using the four-parameter method in Prism. We tested several models (linear, exponential, logistic, and sigmoid) and picked the model that gave the best adjusted r^2^. We used the adjusted r^2^ to account for overfitting. When the curve-fitting program suggested a clearly erroneous result, such as an extremely high top or low bottom, we fixed the top or bottom at the average level for the vigor or for the average of the last three points for the bottom.

### RNAseq

Flies were injected with 50 nL of 0.01 OD600 (1000 CFUs) of *L*. *monocytogenes* resuspended in PBS. Flies were placed in vials containing dextrose fly media and incubated at 29°C. 3 DPI flies were flipped onto dextrose fly media containing 1 mg/mL ampicillin. Groups of 20 flies were homogenized in TRIzol for RNA extraction on the following DPI: 0, 1, 2, 3, 4, 6, 8, 10, 12, 14, 16, and 18. Additional flies were used to determine CFU and monitor survival. We tested w^1118^, *CG2247*, *Pcmt*, and RAL359 strains. As a control, additional sets of w^1118^ were left uninfected and flipped onto dextrose fly media containing 1 mg/mL ampicillin 3 dpi, and samples for RNAseq were collected at the indicated time points. RNA was isolated using standard TRIzol preparation. Library preparation and sequencing was performed by the Duke Center for Genomic and Computational Biology. Briefly, quality of RNA was assessed by Bioanalyzer 2100. Polyadenylated RNA was enriched from total RNA. Barcoded TruSeq cDNA Libraries were constructed and quality was assessed by Qubit and Agilent Tapestation. 50 bp single end reads were obtained by sequencing the libraries on an Illumina HiSeq 2000 using a full flow cell. Reads were mapped using STAR, and RPKM for each gene was determined by Cufflinks.

### Melanization Assay

Groups of 20 female flies were anesthetized with carbon dioxide and injected with 50 nl of 0.01 OD600 (~1,000 CFUs) of *L*. *monocytogenes* resuspended in PBS. After injection, flies were flipped onto dextrose fly media and incubated at 29°C. At 2 dpi, the percent melanized for each group was determined by anesthetizing the flies with carbon dioxide and scoring each abdomen for presence of melanization spots. For each genotype, eight groups of flies were scored.

## Supporting Information

S1 Data Data used to plot the following figures: Figs 2A–2C, 3A–3D and S6A–S6C. (XLSX)

S2 Data Data used to plot the following figures: Figs 4–6 and S1–S5. (XLSX)

S1 Fig Heat map depiction of recovery microarray experiment. All differentially expressed genes were hierarchically clustered. Values are plotted as log2 fold changes relative to the median level of samples collected at 0 DPI. Blues are genes that have lower relative expression, and reds are genes that have higher relative expression.
(PNG)

S2 Fig 50 fuzzy c-means clusters. All differentially expressed genes were clustered into 50 fuzzy c-means clusters. Prior to clustering, data were standardized to have a mean value of zero and a standard deviation of one. The 50 fuzzy c-means groups were then manually curated into groups of similar expression patterns. Each line represents mean gene expression. Blue represents uninfected control. Red represents infected and no treatment. Green represents infected and ampicillin-treated. Cluster numbers highlighted in red contain mortality genes, and cluster numbers highlighted in green contain recovery genes.
(TIF)

S3 Fig Variation of tolerance curves. Tolerance curves showing variation in EC_50_ are shown in (A). (B) shows tolerance curves for flies with reduced resistance. (C) shows tolerance curves for flies in which vigor has changed from the wild-type flies. Data are reported in S2 Data.
(EPS)

S4 Fig Full data for individual tolerance curves of mutant and aged flies. Data are reported in S2 Data.
(EPS)

S5 Fig Full data for individual tolerance curves of *D*. *melanogaster* Genetic Resource Collection flies. Data are reported in S2 Data.
(EPS)

S6 Fig *CG2247* mutants have impaired melanization response during Listeria infection. (A and B) RNAseq data for the phenol oxidases proPOA1 and proPO 45 showing that transcript levels drop in wild-type flies and recover upon antibiotic treatment at 2 DPI, while the *CG2247* mutant does not. (C) reports the amount of melanization seen in infected flies 2 DPI (unpaired *t* test, *p* < 0.0001).
(EPS)

S1 Table Ayasdi dataset. (XLSX)

S2 Table 50 fuzzy c-means clusters. Differentially expressed genes were separated into 50 fuzzy c-means groups. Each fuzzy c-means group is labeled 1–50.
(XLSX)

S3 Table Significantly enriched gene ontology groups. (XLSX)

S4 Table Parameter description for mathematical model. (XLSX)

S5 Table Statistical parameters from the analysis of sigmoid curves in Figs 5 and 6. (XLSX)

## Abbreviations

ActA: actin assembly-inducing protein
AMP: antimicrobial peptide
CFU: colony-forming unit
DPI: days post infection
EC_50_: half maximal effective concentration
LLO: listeriolysin O
MTD: median time to death
PBS: phosphate buffered saline
TDA: topological data analysis
