# Correction: How Many Parameters Does It Take to Describe Disease Tolerance?

**DOI:** 10.1371/journal.pbio.1002485

**Published:** 2016-06-06

**Authors:** The PLOS Biology Staff

## Notice of Republication

This article was republished on May 19^th^, 2016 to correct poor figure quality in the PDF version, which was introduced during the typesetting process. The publisher apologizes for these issues with the figure quality. Please download this article again to view the updated version.

## References

[1] Louie A, Song KH, Hotson A, Thomas Tate A, Schneider DS (2016) How Many Parameters Does It Take to Describe Disease Tolerance? PLoS Biol 14(4): e1002435 doi:10.1371/journal.pbio.1002435 27088212 10.1371/journal.pbio.1002435 PMC4835111

