# Supplementary figures and images for: How Many Parameters Does It Take to Describe Disease Tolerance?
Source: PLoS Biol. 2016 Apr 18;14(4):e1002435. doi: 10.1371/journal.pbio.1002435 (PMC4835111; doi:10.1371/journal.pbio.1002435)

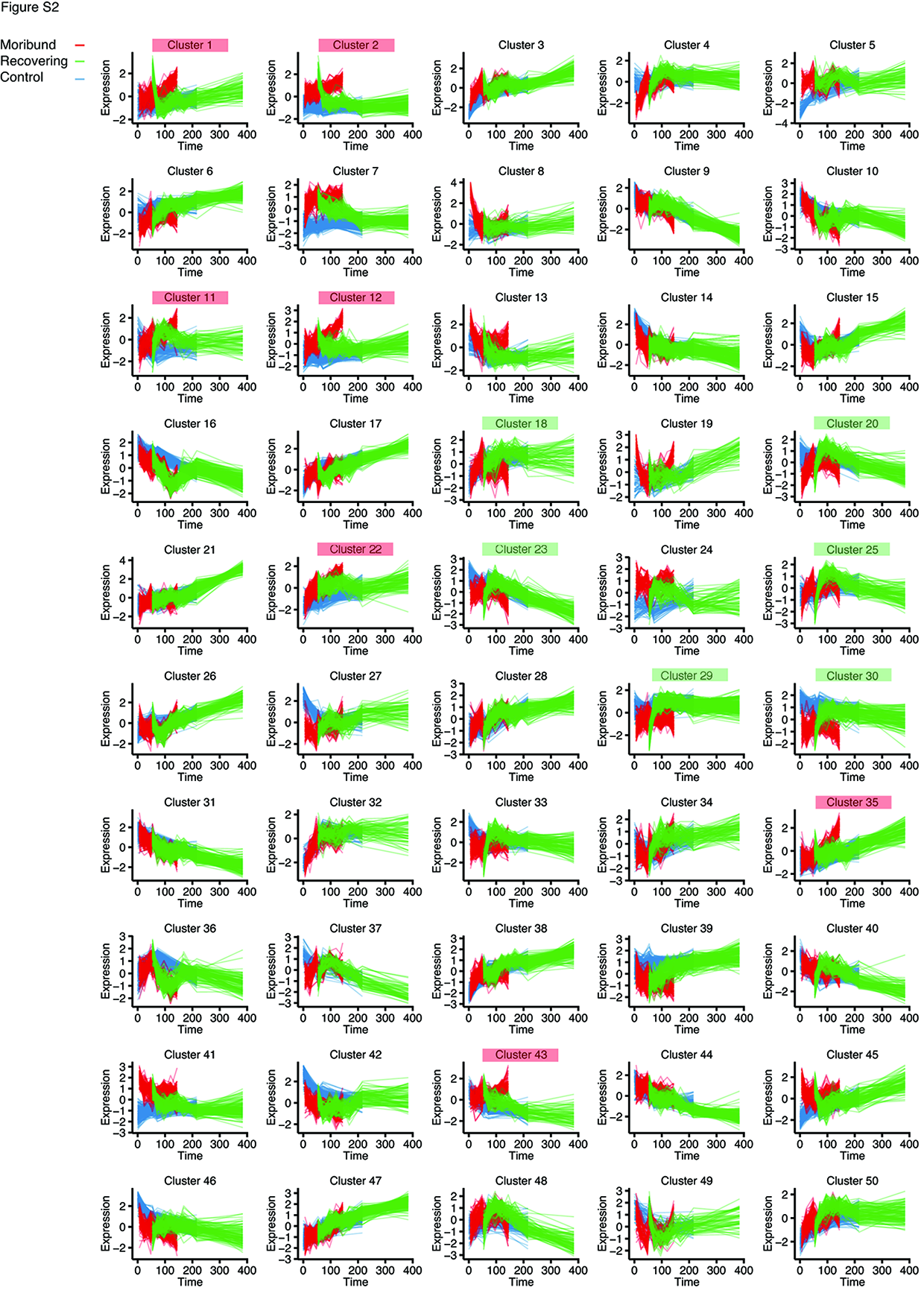

Supplement: S2 Fig — All differentially expressed genes were clustered into 50 fuzzy c-means clusters. Prior to clustering, data were standardized to have a mean value of zero and a standard deviation of one. The 50 fuzzy c-means groups were then manually curated into groups of similar expression patterns. Each line represents mean gene expression. Blue represents uninfected control. Red represents infected and no treatment. Green represents infected and ampicillin-treated. Cluster numbers highlighted in red contain mortality genes, and cluster numbers highlighted in green contain recovery genes. (TIF) [file pbio.1002435.s004.tif]
